# Familial ALS-associated *SFPQ* variants promote the formation of SFPQ cytoplasmic aggregates in primary neurons

**DOI:** 10.1098/rsob.220187

**Published:** 2022-09-28

**Authors:** Jocelyn Widagdo, Saumya Udagedara, Nishita Bhembre, Jing Zhi Anson Tan, Lara Neureiter, Jie Huang, Victor Anggono, Mihwa Lee

**Affiliations:** ^1^ Clem Jones Centre for Ageing Dementia Research, Queensland Brain Institute, The University of Queensland, Brisbane, Queensland 4072, Australia; ^2^ Department of Biochemistry and Chemistry, La Trobe Institute for Molecular Science, La Trobe University, Melbourne, Victoria 3086, Australia

**Keywords:** amyotrophic lateral sclerosis, protein aggregation, DBHS proteins, glutamate receptors, RNA binding proteins, zinc

## Abstract

Splicing factor proline- and glutamine-rich (SFPQ) is a nuclear RNA-binding protein that is involved in a wide range of physiological processes including neuronal development and homeostasis. However, the mislocalization and cytoplasmic aggregation of SFPQ are associated with the pathophysiology of amyotrophic lateral sclerosis (ALS). We have previously reported that zinc mediates SFPQ polymerization and promotes the formation of cytoplasmic aggregates in neurons. Here we characterize two familial ALS (fALS)-associated *SFPQ* variants, which cause amino acid substitutions in the proximity of the SFPQ zinc-coordinating centre (N533H and L534I). Both mutants display increased zinc-binding affinities, which can be explained by the presence of a second zinc-binding site revealed by the 1.83 Å crystal structure of the human SFPQ L534I mutant. Overexpression of these fALS-associated mutants significantly increases the number of SFPQ cytoplasmic aggregates in primary neurons. Although they do not affect the density of dendritic spines, the presence of SFPQ cytoplasmic aggregates causes a marked reduction in the levels of the GluA1, but not the GluA2 subunit of AMPA-type glutamate receptors on the neuronal surface. Taken together, our data demonstrate that fALS-associated mutations enhance the propensity of SFPQ to bind zinc and form aggregates, leading to the dysregulation of AMPA receptor subunit composition, which may contribute to neuronal dysfunction in ALS.

## Graphical abstract

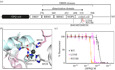

## 1. Introduction

The cytoplasmic aggregation and mislocalization of RNA-binding proteins (RBPs) are emerging hallmarks of neurodegenerative diseases including amyotrophic lateral sclerosis (ALS) [1,2]. These RBPs are best exemplified by trans-activation response element DNA-binding protein 43 (TDP-43) and fused in sarcoma (FUS), both of which predominantly function in the nucleus under normal conditions but are mislocalized and aggregated in the disease state [2,3]. Recent evidence has also demonstrated abnormal cytoplasmic accumulation and loss of the nuclear pool of an RBP, splicing factor proline- and glutamine-rich (SFPQ) protein, in ALS [4–6]. However, the precise molecular mechanisms that underpin these pathological changes, and their effects on neuronal functions are not well understood.

**Figure 1.**
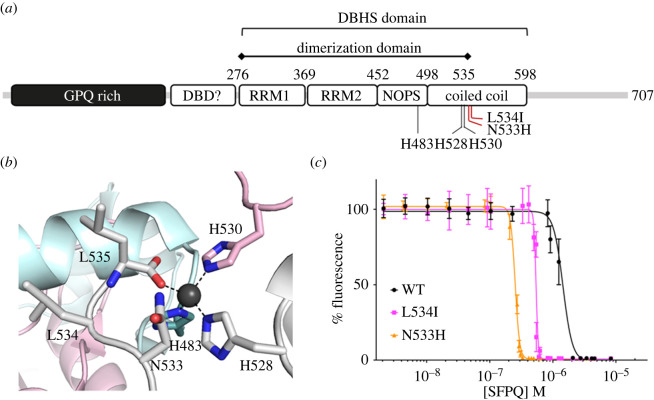
SFPQ N533H and L534I bind to zinc with higher affinities than the WT protein. (*a*) Schematic domain organization of human SFPQ, depicting the positions of the zinc-coordinating residues (H483, H528, and H530) and the reported fALS-associated mutations, N533H and L534I. (*b*) Crystal structure of human SFPQ with a close-up view of the zinc-coordinating centre (PDB code 6OWJ [12]) with the side chains of fALS-associated mutation residues (N533 and L534) displayed in stick presentation. (*c*)The N533H and L534I fALS-associated SFPQ mutants display higher zinc-binding affinities measured by the Zn^2+^ indicator, Fluozin-3 compared to the WT protein. The purified SFPQ DBHS domain (residues 276–598; GST-tag removed) was used for this analysis. Data represent mean ± s.d. from three independent experiments.

SFPQ is an RNA- and DNA-binding protein that is involved in many aspects of RNA biogenesis as well as DNA damage repair [7,8]. It is ubiquitously expressed in most tissues and cell types and has been implicated in a wide range of physiological functions, including neuronal development [7,9–11]. SFPQ belongs to the Drosophila behaviour human splicing (DBHS) protein family together with two paralogs, non-POU domain-containing octamer-binding protein (NONO) and paraspeckle component 1 (PSPC1). The DBHS family proteins share a high sequence similarity within the central DBHS domain which comprises two RNA-recognition motifs (RRMs), a NonA/paraspeckle (NOPS) domain and a long coiled-coil domain (figure 1*a*). It has previously been shown that SFPQ homo- and hetero-dimerizes via the central DBHS domain and polymerizes via the extended coiled-coil domain, which is critical for its nuclear functions, such as transcriptional regulation and paraspeckle formation [13–15]. Although SFPQ primarily functions within the nucleus, there is increasing evidence for cytoplasmic functions of SFPQ, which include the regulation of neuronal RNA transport [16–18]. These observations indicate that the correct balance in the nucleocytoplasmic distribution of SFPQ is critical for neuronal development and homeostasis.

Altered metal homeostasis and increased oxidative stress have consistently been proposed as central features of neurodegeneration [19,20]. Zinc is a transition metal that is selectively stored in, and released from, the presynaptic vesicles of some neurons, with its dysregulation exerting detrimental effects on these cells [20]. We recently discovered that zinc binds to SFPQ and induces SFPQ polymerization *in vitro,* as well as promoting the formation of SFPQ cytoplasmic aggregates in primary neurons [12]. This study supports the notion that the dysregulation of zinc in the brain triggers an imbalance in the nucleocytoplasmic distribution of SFPQ, with several studies reporting an elevated level of zinc in the serum and cerebrospinal fluid of ALS patients [21–23].

Here, we focused on two recently identified missense mutations of SFPQ in familial ALS (fALS; N533H and L534I), both of which cause morphological abnormalities in the axons of motor neurons in the zebrafish [5]. Interestingly, these mutations are located in close proximity to the intermolecular zinc-coordinating centre of SFPQ, which is composed of His-483, His-528, His-530 and the carbonyl oxygen of Leu-535 [12]. We therefore tested the hypothesis that these fALS-associated mutations enhance the zinc-binding affinity of SFPQ, thereby increasing its propensity to aggregate in the cytoplasm of disease-affected neurons. We also investigated the effects of SFPQ N533H and L534I mutants on the structure and function of excitatory synapses by examining the density of dendritic spines and the levels of surface α-amino-3-hydroxy-5-methyl-4-isoxazoleproprionic acid (AMPA)-type glutamate receptors (AMPARs) in primary rat cortical neurons.

## 2. Results

### 2.1. SFPQ N533H and L534I display higher zinc-binding affinities than WT protein

We have previously discovered that SFPQ directly binds to zinc, which promotes the formation of infinite polymers of both the SFPQ homodimers and SFPQ/NONO heterodimers [12]. The intermolecular zinc-coordinating centre within the SFPQ DBHS domain comprises His-483, His-528, His-530, and the carbonyl group of Leu-535 (figure 1*a,b*). The identification of two fALS-associated *SFPQ* missense variants in the proximity of the SFPQ zinc-coordinating centre (N533H and L534I) suggests that they may affect the affinity of SFPQ to zinc. In particular, the additional histidine residue in the N533H mutant may be involved in zinc coordination, thereby enhancing the affinity of SFPQ to zinc. To test this hypothesis, we performed a competitive zinc-binding assay on the purified SFPQ DBHS domain (residues 276–598; figure 1*a*) containing the N533H or L534I mutations using the fluorescent zinc indicator, Fluozin-3 [12]. It is important to note that the zinc-binding affinity of full-length DBHS domain (residues 276–598) is similar to that of truncated dimerization domain (residues 276–535) used for protein crystallization in this study [12]. The calculated affinity (IC_50_) of SFPQ N533H (0.25 µM) and L534I (0.54 µM) binding to zinc was approximately 5.8- and 2.7-fold higher than that of the WT protein (1.45 µM), respectively (figure 1*c*; electronic supplementary material, table S1), confirming that these fALS-associated mutations cause significant increases in SFPQ zinc-binding affinity. Although the effect of SFPQ N533H was expected, it was surprising to observe the increased zinc-binding affinity for the L534I mutant given the subtle nature of the substitution. It is also noteworthy that the slope of fluorescence reduction was significantly steeper for SFPQ L534I than SFPQ WT, whereas that of SFPQ N533H was modest, represented by Hill slopes of −25.1 (L534I), −12.4 (N533H), and −5.9 (WT) (figure 1*c*; electronic supplementary material, table S1). The crystal structure of SFPQ L534I in complex with zinc described below partly explains this cooperativity.

### 2.2. The crystal structure of SFPQ L534I reveals a second zinc-binding site

**Figure 2.**
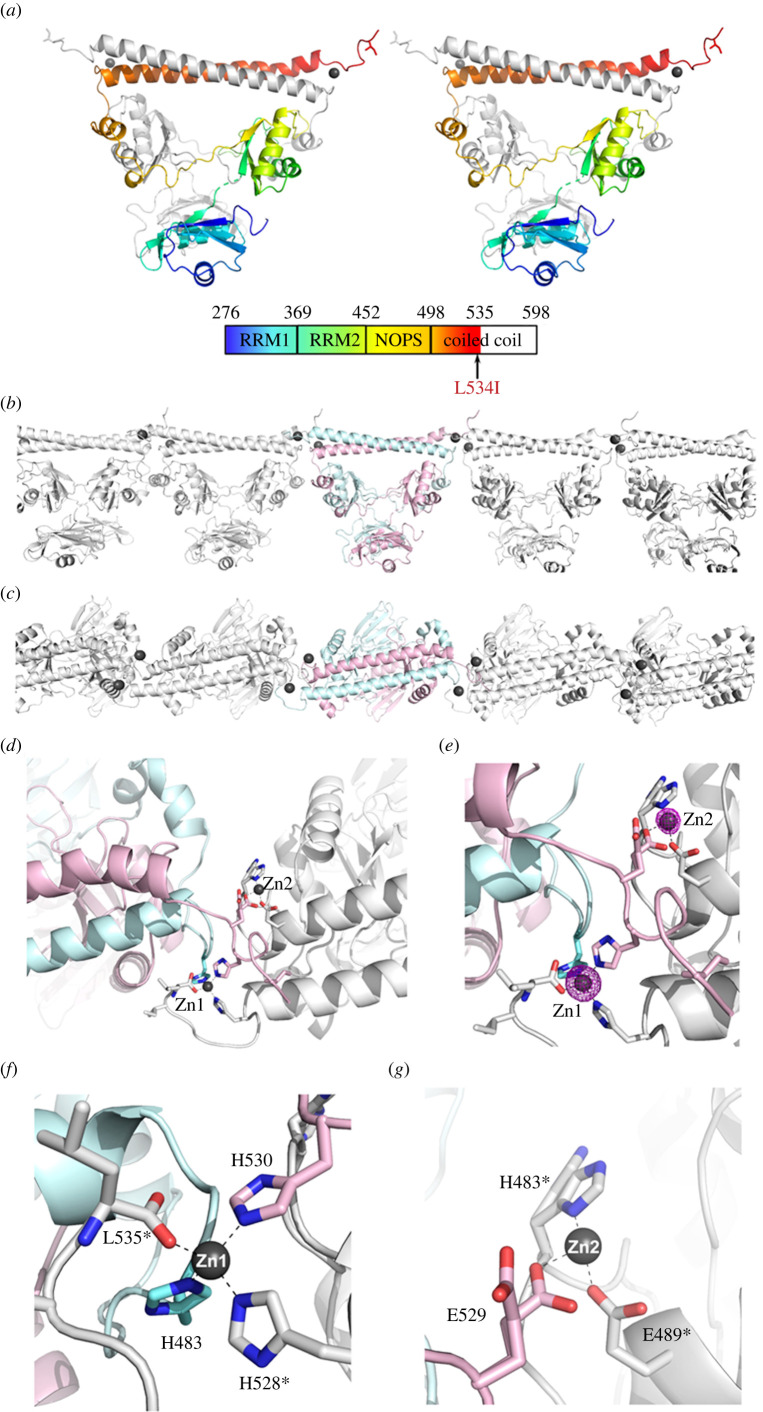
The crystal structure of the SFPQ L534I mutant reveals a second zinc-binding site. (*a*) Stereo view of the dimerization domain of the human SFPQ (residues 276–535) L534I mutant in complex with zinc in cartoon presentation with Chain A in grey and Chain B in the same colour scheme as in the schematic domain organization below. The Zn atoms are shown as black spheres. (*b*,*c*) Zinc-mediated infinite polymerization observed in the crystal structure of SFPQ L534I from the side (*b*) and top (*c*) views. Chains A and B are shown in cyan and pink, respectively, while the neighbouring symmetry-related dimers are shown in grey. (*d*) Zinc centres in the crystal structure of the SFPQ L534I mutant. The intermolecular interaction mediated by two zinc atoms is shown with Chain A and Chain B in cyan and pink, respectively, while the neighbouring dimer (symmetry operator: *x*, *y*, *z* – 1) is shown in grey. (*e*) Close-up view of the zinc centres with overlaid anomalous difference Fourier map (|*F*^+^|–|*F*^−^|, contoured at 7 *σ*) from the diffraction data collected at 9760 eV near the Zn X-ray absorption edge (*f″*_Zn_ = 3.77 e^−^) for zinc identification. The positions of the L534I mutation are shown with the side chain of Ile in stick presentation. (*f*) Zinc centre 1 (Zn1) is coordinated by H483 (Chain A), H530 (Chain B), H528* (Chain A of neighbouring dimer), and carbonyl oxygen of L535* (Chain A of neighbouring dimer). The asterisk (*) denotes a symmetry-related dimer (*x*, *y*, *z* – 1). (*g*) The partially occupied zinc centre 2 (Zn2; occupancy of 0.5) is coordinated by E529 (Chain B), H483* and E489* (Chain B of neighbouring dimer).

To gain insights into the structural basis of the enhanced zinc-binding affinity of the fALS-associated SFPQ mutant, we solved the crystal structure of the SFPQ dimerization domain (residue 276–535) containing the L534I mutation in complex with Zn(II) (figure 2*a*). The approach to produce crystals of SFPQ WT in complex with Zn(II) was the same as that used previously [12]. Briefly, the DNA-binding domain of retinoic X receptor *α* (RXR*α*), which contains two zinc finger domains, was incubated with the SFPQ L534I mutant protein (1:1 molar ratio) and crystallized by the hanging-drop vapour diffusion method at 20°C [12]. The resulting crystals of SFPQ L534I in complex with Zn(II) have similar cell dimensions of the WT SFPQ crystals in complex with Zn(II) and the overall structure is similar to that of SFPQ WT with the root mean square deviation (r.m.s.d.) of 0.28 Å between them (496 common C*α* superposed) (table 1; figure 2*a*; electronic supplementary material, figure S1). Consistent with SFPQ WT, the intermolecular interaction mediated by zinc also results in infinite polymerization of SFPQ L534I (figure 2*b*,*c*). Given the subtle nature of the substitution from leucine to isoleucine, these observations were expected. However, the structural refinement of SFPQ L534I revealed a surprising additional metal density, which also mediates the intermolecular interaction of SFPQ (figure 2*b*,*c*). The identity of this metal ion was confirmed as zinc by calculating the anomalous difference Fourier maps from diffraction data collected at energies near the zinc X-ray absorption edge: 9760 eV (*f″*_Zn_ = 3.77 e^−^) and 9560 eV (*f″*_Zn_ = 0.57 e^−^) (figure 2*d*,*e*; electronic supplementary material, figure S2).

**Table 1.** Diffraction data and refinement statistics. Values in parentheses are for the highest-resolution shell.

data collection	
space group	*P*2_1_
unit cell parameters (Å,°)	61.6, 62.7, 67.8, *β* = 96.1
resolution (Å)	45.92–1.83 (1.87–1.83)
no. of observations	151 869 (9690)
no. of unique reflections	44 977 (2803)
completeness (%)	98.9 (99.8)
redundancy	3.4 (3.5)
*R*_merge_ (%)	3.1 (28.4)
*R*_pim_ (%)	2.3 (20.5)
CC_1/2_	0.999 (0.913)
average I/*σ*(I)	17.8 (3.6)
refinement	
*R* (%)	17.8 (23.2)
*R*_free_ (%)	21.6 (27.7)
no. (%) of reflections in test set	2280 (5.1)
no. of protein molecules per asu	2
R.m.s.d bond length (Å)	0.01
R.m.s.d bond angle (°)	1.66
average B-factors (Å^2^)^a^	36.0
protein molecules	35.7
zinc atoms	25.2
water molecules	42.1
Ramachandran plot^b^	
residues other than Gly and Pro in:	
most favoured regions (%)	98.6
additionally allowed regions (%)	1.4
disallowed regions (%)	0
pdb code	7SP0

^a^Calculated by BAVERAGE in CCP4 Suite [24].

^b^Calculated using MolProbity [25].

A closer inspection of the two zinc centres showed that the first zinc coordinating centre (Zn centre 1) is almost indistinguishable from that of SFPQ WT, coordinated by His A483, His B530, His A*528 and a carbonyl oxygen of Leu A*535, where the asterisk (*) denotes a symmetry-related dimer (*x*, *y*, *z* – 1) (figure 2*f*). The Zn^2+^ ion in this centre is fully occupied with a comparable *B*-factor value (22.4 Å^2^) to those of ligating atoms (average *B*-factor of 20.9 Å^2^). However, the anomalous signal from the second centre (Zn centre 2) is significantly lower than that of Zn centre 1 (figure 2*e*), indicating that this site is not fully occupied. The second zinc coordination centre is composed of three amino acid residues: Glu B*489, Glu B529 and His B*483 (symmetry operator: *x*, *y*, *z* – 1). Further supporting the partially occupied zinc, the side chain of two out of three of these amino acids is also observed in two different conformations (figure 2*g*). The side chains of Glu B529 and His B*483 are in the ‘in’ position coordinating zinc, whereas in the absence of zinc, they take on the ‘out’ conformation. Zinc in the second centre is modelled with the occupancy of 0.5 in the final structure with a comparable *B*-factor value (28.1 Å^2^) to those ligating atoms (average *B*-factor of 26.8 Å^2^). The fourth zinc-ligating atom is likely to be a partially occupied solvent atom; however, it could not be modelled in the final structure due to its low electron density (electronic supplementary material, figure S2). Taken together, the crystal structure of SFPQ L534I in complex with zinc reveals an additional zinc-binding site, providing the structural basis for the apparent increase in zinc-binding affinity in this mutant.

### 2.3. fALS-associated N533H and L534I mutants promote SFPQ cytoplasmic aggregation in neurons

**Figure 3.**
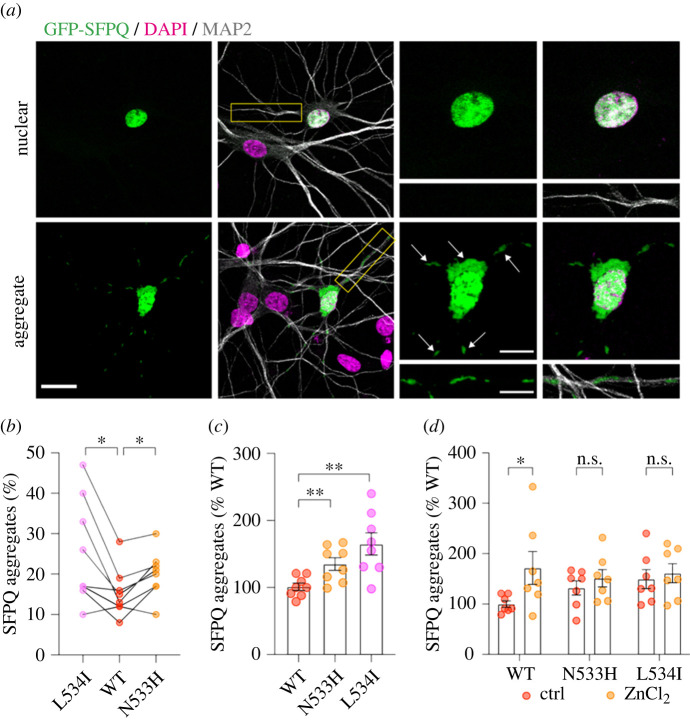
fALS-associated N533H and L534I mutants promote the cytoplasmic aggregation of SFPQ. (*a*) Representative confocal images of primary cortical neurons expressing GFP-SFPQ (green) exhibiting nuclear localization (top panel) or cytoplasmic aggregates in the somatodendritic regions (white arrows, bottom). Higher-magnification images are shown on the right panels. Scale bars, 50 µm or 10 µm (inset). (*b*)Quantification of the fraction of neurons with GFP-SFPQ cytoplasmic aggregates within individual experiments (Wilcoxon matched-pairs *t*-test, *p < 0.05, *N* = 5 independent experiments). (*c*) Quantification of the fraction of neurons with GFP-SFPQ cytoplasmic aggregates normalized to the value of the WT group. Data represent mean ± SEM (Welch's unpaired *t*-test, **p < 0.01, *N* = 5 independent experiments). (*d*) Zinc-induced SFPQ aggregation is occluded in neurons expressing fALS-associated mutants. Primary cortical neurons expressing GFP-SFPQ, either WT, N533H or L534I mutants, were treated with 100 µM ZnCl_2_ for 4 h. Data represent mean ± SEM, normalized to the WT group (two-way ANOVA, Sidak's *post-hoc* multiple comparison test, *p < 0.05, n.s. = not significant, *N* = 3 independent experiments). Each data point was derived from one coverslip, which contained an average of 113 transfected neurons.

Mislocalization and cytoplasmic aggregation of nuclear SFPQ are associated with the pathogenesis of ALS [4–6]. We have previously demonstrated that the application of ZnCl_2_ enhances the cytoplasmic accumulation and aggregation of SFPQ when overexpressed in cultured neurons [12]. To determine the effect of N533H and L534I mutations on SFPQ localization, we transiently transfected primary cortical neurons with DNA constructs that encode GFP-SFPQ, either WT, N533H or L534I for 24 h. Under basal conditions, overexpression of GFP-SFPQ N533H and L534I significantly enhanced the propensity to form cytoplasmic aggregates in primary cortical neurons compared to the WT protein (figure 3*a–c*; electronic supplementary material, figure S3). These effects were not due to alterations in protein levels as GFP-SFPQ N533H and L534I expressed at a comparable level to the WT protein when overexpressed in HEK293T cells (electronic supplementary material, figure S4). As expected, the addition of 100 µM ZnCl_2_ to the culture medium for 4 h significantly increased the number of neurons with cytoplasmic GFP-SFPQ aggregates in the somatodendritic regions (figure 3*d*). However, ZnCl_2_ treatment did not result in a further increase in the proportion of neurons containing GFP-SFPQ cytoplasmic aggregates when transfected with the N533H and L534I mutants, likely because the zinc-binding sites in these mutants have been fully occupied (figure 3*d*). Together, these data indicate that the increase in zinc-binding affinity accounts for the elevation in the number of cytoplasmic aggregates observed in neurons that express these two fALS-associated SFPQ mutants.

### 2.4. Cytoplasmic SFPQ aggregation causes a reduction in the level of surface GluA1 expression

**Figure 4.**
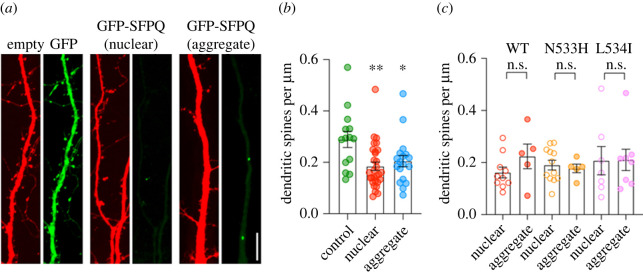
Overexpression of GFP-SFPQ reduces dendritic spine density, a phenotype that is not affected by fALS-associated *SFPQ* variants. (*a*) Confocal images of dendritic segments of neurons co-expressing the structural marker td-Tomato (red) and soluble GFP (left panels) or GFP-SFPQ (green) that exhibit nuclear localization (middle panels) or cytoplasmic aggregates in the somatodendritic regions (right panels). Scale bar, 10 µm (inset). (*b*) Quantification of dendritic spine density from neurons that express soluble GFP, nuclear GFP-SFPQ or GFP-SFPQ aggregates. Data are presented as mean ± SEM (one-way ANOVA, Tukey's *post-hoc* multiple comparison test, *p < 0.05 **p < 0.01; GFP, *n* = 14 neurons; nuclear, *n* = 29 neurons; aggregate, *n* = 18 neurons, *N* = 2 independent experiments). (*c*) Quantification of dendritic spine density from neurons expressing GFP-SFPQ WT, N533H or L534I mutants. Data are presented as mean ± SEM (Welch's unpaired *t*-test, ns = not significant; *N* = 2 independent experiments).

Our observation that SFPQ cytoplasmic aggregates are largely localized in the soma and dendrites raises the question of whether these protein aggregates affect dendritic functions in primary neurons. To investigate this, we first examined the effect of SFPQ overexpression on the density of dendritic spines of neurons that co-expressed a structural marker td-Tomato and GFP or GFP-SFPQ, either WT, N533H or L534I (figure 4*a*; electronic supplementary material, figure S5). Interestingly, overexpression of GFP-SFPQ caused a significant reduction in the number of dendritic spines compared with those expressing soluble GFP alone (figure 4*a*,*b*). This effect was observed in all neurons containing nuclear or aggregated GFP-SFPQ (figure 4*a*,*b*). However, there was no significant difference between GFP-SFPQ WT or fALS mutants (figure 4*c*), suggesting that these mutations play no role in regulating the density of dendritic spines in primary neurons.

**Figure 5.**
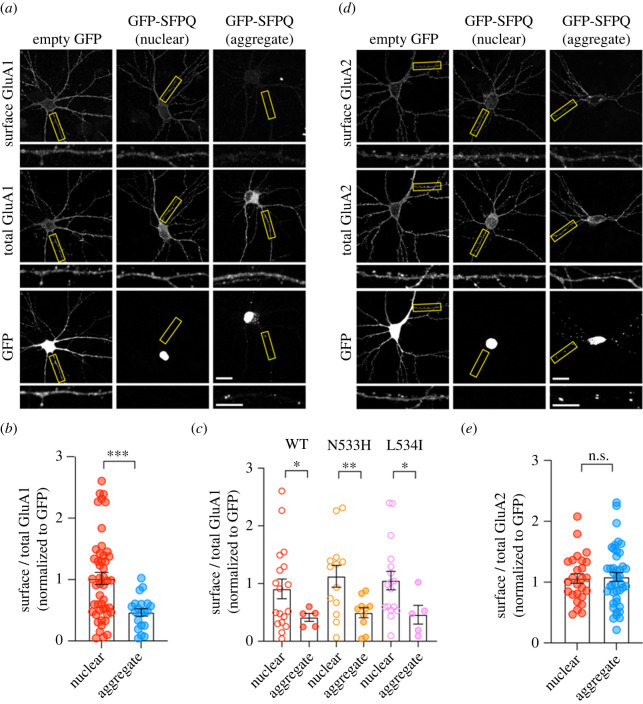
Cytoplasmic SFPQ aggregates selectively reduce the levels of GluA1- but not the GluA2-containing AMPARs on the plasma membrane. (*a*) Primary cortical neurons were co-transfected with plasmids encoding myc-GluA1 and GFP or GFP-SFPQ. Representative images of surface and total myc-GluA1 in neurons expressing soluble GFP, nuclear GFP-SFPQ or GFP-SFPQ cytoplasmic aggregates. Zoomed images are dendrites from the boxed regions. Scale bars, 20 µm or 10 µm (enlarged images). (*b*) Quantification of the surface/total GluA1 ratio normalized to the value of GFP-expressing neurons. Data are presented as mean ± SEM (Welch's unpaired *t*-test, ***p < 0.001; nuclear, *n* = 48 neurons; aggregate, *n* = 20 neurons, from two independent experiments). (*c*) Quantification of the surface/total GluA1 ratio from neurons expressing GFP-SFPQ WT, N533H or L534I mutants, normalized to the value of GFP-expressing neurons. Data are presented as mean ± SEM (Welch's unpaired *t*-test, *p < 0.05 **p < 0.01; *N* = 2 independent experiments). (*d*) Confocal images of primary neurons co-expressing myc-GluA2 and soluble GFP, nuclear GFP-SFPQ or GFP-SFPQ cytoplasmic aggregates. Scale bars, 20 µm or 10 µm (enlarged images). (*e*) Cytoplasmic SFPQ aggregation does not affect the expression of surface GluA2-containing AMPARs. Data represent mean ± SEM (Welch's unpaired *t*-test, n.s. = not significant; nuclear, *n* = 26 neurons; aggregate, *n* = 43 neurons, from two independent experiments).

Given that excitotoxicity and altered cortical excitability are pathophysiological features associated with ALS [26], we next investigated the effect of the N533H and L534I mutants on the surface expression of the GluA1 and GluA2 subunits of AMPARs, which mediate the majority of fast excitatory synaptic transmission in the mammalian central nervous system [27]. Primary neurons were transfected with myc-GluA1 or myc-GluA2 reporter constructs with plasmids that encode GFP or GFP-SFPQ, either WT, N533H or L534I, followed by a surface staining assay with anti-myc antibodies. Neurons that expressed nuclear GFP-SFPQ, regardless of their genotypes, had normal levels of GluA1-containing AMPARs on the plasma membrane compared to those that expressed soluble GFP alone (figure 5*a*,*b*; electronic supplementary material, figure S6). However, cytoplasmic SFPQ aggregates caused a significant decrease in the expression of GluA1-containing AMPARs on the plasma membrane of neurons that overexpressed GFP-SFPQ WT, N533H or L534I **(**figure 5*a*–*c*). This effect was specific to GluA1 as cytoplasmic SFPQ aggregates did not affect the expression of surface GluA2-containing AMPARs in primary neurons (figure 5*d*,*e*). Collectively, our results demonstrate that aberrant cytoplasmic aggregation of SFPQ causes a selective loss of GluA1-containing AMPARs on the neuronal plasma membrane. Given that the fALS-associated *SFPQ* variants that we examined have a higher propensity to promote the formation of SFPQ cytoplasmic aggregates, they may alter neuronal excitability by changing the subunit composition of synaptic AMPARs during the disease progression.

## 3. Discussion

SFPQ is a ubiquitous nuclear RBP that is highly expressed in the brain, with multiple roles in transcriptional regulation, alternative splicing, mRNA transport, paraspeckle formation and RNA metabolism [7,8]. Nuclear depletion and cytoplasmic accumulation of SFPQ have been linked to the pathophysiology of neurodegenerative diseases, including Alzheimer's disease [28] and ALS [4,5]. Despite this, our understanding of how SFPQ mislocalizes into the cytoplasm and forms protein aggregates is very limited. Our previous work has demonstrated the role of zinc in inducing SFPQ polymerization *in vitro* and the formation of SFPQ cytoplasmic aggregates in primary neurons [12]. In the present study, we characterize two fALS-associated *SFPQ* variants that result in amino acid substitutions (N533H and L534I) in the proximity of the SFPQ zinc-coordinating centre. We found that both mutants enhance zinc-binding affinity and the propensity to form cytoplasmic aggregates in the somatodendritic region of primary neurons.

### 3.1. Structural basis of enhanced zinc-binding affinity in SFPQ L534I

The identification of a second zinc-binding site in the SFPQ L534I structure explains the apparent increase in zinc-binding affinity. It also explains the higher Hill slope measured in this variant in comparison to the WT protein. Given that there is more than one zinc-binding site in SFPQ L534I, we fitted the data from the zinc-binding assays with the IC_50_ model instead of calculating K_d_ values using the one site nonlinear fit in our previous study with SFPQ WT [12]. It is noteworthy that the addition of the DNA-binding domain (DBD) of RXR*α* was critical for the crystallization of SFPQ L534I in a complex with Zn(II). Although direct and instant zinc transfer from RXR*α* to SFPQ was not observed when subjected to inductively coupled plasma mass spectrometry (ICP-MS; data not shown) as expected owing to the higher zinc-binding affinity of zinc-finger domains (0.1 pM to 10 nM), it is speculated that slow release of Zn(II) from RXR*α* presumably due to gradual protein degradation and/or oxidation of Zn centre in the zinc finger domain of RXRα-DBD may have facilitated crystallization of the Zn–SFPQ complex over 1–2 weeks at 20°C. In addition, excess Zn(II) were present in the crystallization: 2 Zn(II) per RXRα-DBD mixed with 1:1 molar ratio with SFPQ monomer, and therefore 4 Zn(II) available for SFPQ dimer theoretically. However, the condition used for crystallization was the same as in the WT SFPQ crystallization from our previous study. This raises the question as to which structural changes accommodate the second zinc-binding site in this variant. Leu-534 is not involved either in the dimerization interface or direct polymerization interface. Therefore, it is unlikely that this mutation would impact the dimerization or polymerization status of SFPQ, which has the potential to alter the Zn^2+^-binding affinity. An overall structural comparison between the L534I mutant with SFPQ WT in complex with zinc reveals no obvious differences that can explain the increased zinc-binding of SFPQ L534I (electronic supplementary material, figure S1). The main structural deviations are concentrated at the end of the N-terminal region upstream of the structured RRM1 due to a lack of crystal contacts. Part of the NOPS domain in Chain A (residues 449–477) shows higher r.m.s.d. values than the mean value of 0.28 Å with the greatest C*α* r.m.s.d. of 1.0 Å (Met-469). However, how these subtle displacements increase the Zn^2+^-binding affinity of SFPQ L534I is not clear.

This led us to revisit the crystal structure of SFPQ WT in complex with zinc. The anomalous difference Fourier maps calculated from the diffraction data collected at the high Zn energy wavelength at 9760 eV clearly show the difference between WT and the L534I mutant (electronic supplementary material, figure S7). In the position corresponding to Zn centre 2, there is a small anomalous signal that accounts for a possible weakly bound Zn(II) in SFPQ WT. However, it is significantly weaker than that of SFPQ L534I, reinforcing our observation that this mutation enhances the zinc-binding affinity of SFPQ in solution. The final model of SFPQ WT has a water molecule in this site with a *B*-factor value of 29.5 Å^2^, while O*ε*1 of Glu-489, a potential hydrogen donor to this water molecule, has a comparable *B*-factor value of 23.3 Å^2^. Although it is subtle, the substitution of leucine to isoleucine may affect the dynamics of SFPQ in the solution, either increased dynamics causing the increased on-rate or decreased dynamics reducing the off-rate of zinc-binding events, thereby facilitating the increased zinc-binding affinity. However, the precise underlying molecular mechanism requires further investigation.

At present, we are not able to ascertain if the additional histidine in SFPQ N533H is indeed replacing the carbonyl oxygen atom of Leu-535 in the zinc-coordinating centre in the absence of the zinc-bound SFPQ N533H crystal structure. However, the fact that SFPQ N533H (0.25 µM) displays a higher zinc-binding affinity than SFPQ L534I (0.54 µM), and the failure to crystallize this mutant under the same conditions as SFPQ WT and L534I, may indicate that SFPQ N533H has a different zinc-coordinating centre.

### 3.2. Cytoplasmic SFPQ aggregates selectively reduce the expression of GluA1-containing AMPARs

Excitotoxicity is a major cause of neuronal death in several neurodegenerative conditions, including ALS [29], with altered cortical excitability and synaptic dysfunction being identified as early pathophysiological features in this disease [26,30]. Emerging evidence has demonstrated attenuated synaptic function in neurons expressing ALS-associated mutant proteins, particularly alterations in their dendritic arbor complexity, dendritic spine density and the level of postsynaptic ionotropic AMPARs [31–33]. We found that overexpression of GFP-SFPQ significantly downregulates the number of dendritic spines in primary cortical neurons compared to those that express soluble GFP. Interestingly, this effect was observed in all neurons with nuclear or cytoplasmic SFPQ aggregates. One plausible mechanism may involve the sequestration of an important SFPQ interacting partner FUS, leading to its loss of function and consequently, a deficit in the dendritic spine morphogenesis [34–36]. Alternatively, overexpression of SFPQ can dysregulate the transcription of long genes, many of which are involved in neuronal development and neurite outgrowth [11]. Although SFPQ overexpression-induced loss of dendritic spines is not affected by fALS-associated *SFPQ* variants, our data suggest that SFPQ is an important regulator of dendritic spine formation and/or maturation in excitatory neurons, a finding which warrants further investigation.

AMPARs are assembled as two identical heterodimers of GluA1-4 subunits that form functional glutamate-gated ion channels. The presence of the GluA2 subunit renders AMPARs impermeable to Ca^2+^. Alterations in AMPAR subunit composition have been reported in human post-mortem tissues of ALS patients [37,38] and in various ALS models that overexpress ALS-associated proteins, including TDP-43, FUS and C9ORF72 [34,39–41]. Consistent with this notion, we found that neurons that contain cytoplasmic SFPQ aggregates have significantly reduced surface expression of GluA1-containing AMPARs compared to those that express nuclear SFPQ. This effect is specific to the GluA1 subunit as the aggregation of SFPQ does not affect the GluA2 subunit. Interestingly, the two fALS-associated *SFPQ* variants do not directly contribute to altering AMPAR subunit composition in neurons *per se* given that their effects are indistinguishable from those that overexpress SFPQ WT.

ALS-associated alterations in AMPAR subunit composition can occur due to dysregulation of *GRIA* transcripts or inefficient RNA editing of *GRIA2* mRNA that affects AMPAR permeability to Ca^2+^ [29,42]. However, we posit that cytoplasmic SFPQ aggregates are likely to perturb the trafficking of GluA1-containing AMPARs in primary neurons. Our hypothesis is supported by the fact that SFPQ is present in AMPAR-containing vesicles purified from mouse whole brain lysates [43], and the demonstration that it also interacts with the motor protein KIF5 [18], which has previously been shown to regulate the dendritic transport of such vesicles [44–47]. Importantly, mutations in the KIF5A C-terminal cargo binding domain are associated with fALS [48,49]. This highlights that disruption in KIF5-mediated transport of not only RNA granules but also neurotransmitter receptors contributes to the pathogenesis of ALS. Although our findings are consistent with the general idea of a perturbation of AMPAR subunit expression as a feature of ALS pathology, cytoplasmic SFPQ aggregates may not cause an increase in the expression of GluA2-lacking Ca^2+^-permeable AMPARs, at least in cortical neurons, that are thought to mediate the excessive Ca^2+^ influx and death of motor neurons [40,50,51]. However, it remains to be determined whether a similar effect is observed in motor neurons, given that the heterogeneity of AMPAR subunit expression has been observed in different regions of the brain and might be dependent on the genetic causes of the disease [37]. Although the exact mechanism of action remains unknown, it is clear from our study that intracellular SFPQ aggregates negatively impact the trafficking of AMPARs, and thus neuronal functions that are likely to be manifested during disease progression.

In conclusion, our current study provides the structural basis to explain the increased propensity of fALS-associated *SFPQ* variants to form cytoplasmic aggregates through enhanced zinc-binding affinity, leading to a loss in SFPQ function in neurons. Although it is well established that genetic variants (familial or sporadic) are the main components that predispose (or cause) an individual to develop ALS [52], the molecular mechanisms underlying pathogenesis and disease progression are likely to differ, depending on the mutations. The *SOD1* gene, which encodes the Cu^2+^/Zn^2+^ superoxide dismutase 1 that protects neurons from oxidative stress, is often mutated in fALS patients. Mechanistically, ALS-associated mutations alter the metal binding capacity of SOD1, leading to protein misfolding and aggregation. Accordingly, delivering zinc to mutant SOD1 exerts protective therapeutic effects in the SOD1-G37R ALS mouse model [53]. On the contrary, our findings predict that zinc supplementation would exacerbate SFPQ aggregation and therefore have negative impacts on neuronal function in patients carrying the N533H or L534I mutation. Thus, the outcomes of our study highlight the potential importance of personalized medicine—different mutations may require different interventions—in the treatment of ALS patients with distinct genetic variants.

## 4. Material and methods

### 4.1. DNA constructs

The construction of pCDF11-SFPQ-276–535, pGEX6p1-SFPQ-276–598 and pGEX6p1-RXRα-DBD (residues 130–228) has been described elsewhere [12,14]. The N533H and L534I mutations in the pEGFP-SFPQ and pGEX6P1-SFPQ-276–598 constructs were generated with the Q5 site-directed mutagenesis kit (New England Biolabs). All constructs were verified by DNA sequencing. Plasmid DNAs encoding the myc-tagged GluA1 and GluA2 subunits of AMPARs were gifts from Prof. Richard Huganir and have been described previously [54,55].

### 4.2. Protein expression and purification

His_6_-tagged SFPQ-276–535 L534I was expressed and purified by the method previously reported for wild-type (WT) His-tagged SFPQ-276-535 [14]. His_6_-tag was removed from SFPQ-276-535 L534I and used for crystallization. The procedures for the expression and purification of GST-tagged RXRα-DBD-130–228 (encoding amino acid residues 130–228 of human RXR*α*) were performed according to a previous report [56]. GST-tagged SFPQ constructs (SFPQ-276-598 WT, SFPQ-276-598 N533H and SFPQ-276-598 L534I) were expressed and purified by the method previously reported for GST-SFPQ-276-598 WT [12]. Following the cleavage of the GST-tag, recombinant proteins were then eluted from a HiLoad 16/600 Superdex 200 pg column (GE Healthcare) equilibrated with 20 mM Tris-HCl (pH 7.5), 300 mM NaCl. Final protein samples were concentrated to 4–8 mg ml^−1^, snap-frozen using liquid nitrogen and stored at −80°C. GST-tag was removed from SFPQ constructs and used for the zinc-binding assay.

### 4.3. Zinc-binding assay

Due to the formation of infinite polymers and protein precipitation upon zinc binding, we were not able to measure the binding affinity of SFPQ to zinc directly [57]. Instead, measurements of the zinc-binding affinities of the SFPQ-276-598 WT and mutant proteins (SFPQ-276-598 N533H and SFPQ–276-598 L534I) were achieved with the Fluozin-3 competitive zinc-binding assay as previously described [12,58]. Briefly, competition by ethylenediaminetetraacetic acid (EDTA)-treated SFPQ proteins (GST-tag removed) in 20 mM MOPS (pH 7.0), 250 mM NaCl for Zn(II) binding was assessed by monitoring the decrease in the fluorescence of 150 nM Fluozin-3-Zn(II) with an excitation wavelength of 485 nm and emission wavelength of 520 nm in response to increasing SFPQ protein concentrations. The data were analysed using the equation, log_10_[inhibitor] versus response–variable slope, in Prism (GraphPad Software) to determine the IC_50_ value for zinc-binding.

### 4.4. Crystallization and X-ray diffraction data collection

Crystals of the Zn-SFPQ-276-535 L534I complex were grown using the same strategy as for SFPQ WT 276-535 in complex with Zn(II). Briefly, SFPQ-276-535 L534I (His_6_-tag removed; 2.5 mg ml^−1^) and RXRα-DBD-130-228 (1 mg ml^−1^), both in 20 mM Tris-HCl (pH 7.5), 500 mM NaCl were mixed at a molar ratio of 1:1 and incubated for one hour before being concentrated to 15 mg ml^−1^. The crystals were grown using the hanging-drop vapour diffusion method at 20°C by mixing 2 µl of SFPQ-RXRα-DBD (7.5 mg ml^−1^) with 2 µl of reservoir solution [0.1 M MES (pH 6.0), 0.2 M calcium chloride and 12% (w/v) PEG 4000] and equilibrating against 0.5 ml reservoir solution. Before cryo-cooling, crystals were successively transferred to artificial reservoir solutions containing 20% ethylene glycol in 10% increments. Diffraction data were recorded on beamline MX2 at the Australian Synchrotron [59] at a wavelength of 0.954 Å at 100 K. Additional datasets were collected for metal identification at low- and high-energy remote wavelengths of 9560 eV and 9760 eV, respectively, at 100 K. The data were processed with XDS [60], and merged and scaled with AIMLESS [61]. Crystals belong to space group *P*2_1_ with unit cell parameters of *a* = 61.6, *b* = 62.7, *c* = 67.8 Å and *β* = 96.1°, similar to those of the WT crystals [12]. Data collection and merging statistics for the native dataset are summarized in table 1 and those for the datasets collected for metal identification are in electronic supplementary material, table S2.

### 4.5. Structure solution and refinement

The crystal structure was refined using the Zn-SFPQ complex structure (PDB code 6OWJ [12]) as the initial model after removing all non-protein atoms and mutating L534 to alanine. Iterative model building with COOT [62] and refinement with REFMAC5 [63] within the CCP4 suite [24] was carried out. The final model consisted of two chains of SFPQ (residues 283–535 in Chain A and residues 290–368, 371–535 in Chain B), two Zn atoms (one of the two with half occupancy), and 277 water molecules. The quality of the model was validated using MOLPROBITY [25]. The refinement statistics are included in table 1. The atomic coordinates have been deposited in the Protein Data Bank as entry 7SP0.

### 4.6. Zinc treatment and SFPQ localization assay

Primary cortical neurons were prepared from embryonic day 18 rat pups as described previously [64]. All animal handling procedures were carried out in accordance with the Australian Code of Practice for the Care and Use of Animals for Scientific Purposes and were approved by the University of Queensland Animal Ethics Committee (AEC approval number QBI/047/18). Neurons were transfected at days *in vitro* (DIV) 13 using Lipofectamine 2000 (Invitrogen). The next day, they were treated with either 100 µM ZnCl_2_ for 4 h or water (vehicle control), and subsequently fixed with 4% paraformaldehyde/4% sucrose solution in PBS. Following extensive washes, the neurons were stained with anti-MAP2 antibody (M3696, Sigma-Aldrich) before mounting them onto glass slides using ProLong Diamond anti-fade mounting medium with DAPI (Invitrogen). Slides were imaged on a Zeiss Axio Imager epifluorescence microscope. The fractions of neurons containing nuclear or cytoplasmic GFP-SFPQ aggregates over total transfected neurons were quantified and normalized to the GFP-SFPQ WT without ZnCl_2_ treatment group.

### 4.7. Dendritic spine analysis

Neurons were co-transfected with the structural marker td-Tomato and pEGFP-SFPQ, either WT, N533H or L534I mutants for 48 h. Fixed neurons were imaged with a 100× oil-immersion objective on an inverted Diskovery spinning disc confocal microscope. Secondary dendrites of co-transfected neurons were randomly imaged as z-stacks with a 0.4 µm step size over a range of approximately 5–10 µm. Spine analysis was performed on raw image stacks (*z*-plane) using ImageJ software. Spine density was calculated as the total number of spines per sum of all measured dendritic lengths for each neuron. All clear protrusions from the dendrite, irrespective of their orientation relative to the imaging plane, were included in the analyses.

### 4.8. Surface staining assay

To determine the level of AMPARs on the plasma membrane of primary neurons, we performed an antibody-feeding assay as previously described [54]. Neurons were co-transfected with pRK5-myc-GluA1 or pRK5-myc-GluA2 with pEGFP alone or pEGFP-SFPQ, either WT, N533H or L534I mutants, for 48 h. Surface AMPARs were labelled by incubating live neurons with mouse anti-myc antibody (MCA2200, BioRad) for 30 min at 4°C prior to 10 min fixation in ice-cold parafix solution (4% paraformaldehyde, 4% sucrose in PBS). Following cell permeabilization (0.25% Triton X-100 in PBS, 10 min) and blocking (10% normal goat serum, 1 h) at room temperature, total myc-GluA1/2 was labelled with rabbit anti-myc antibody (71D10, Cell Signaling Technology) at 4°C overnight. The surface and total myc-GluA1 or myc-GluA2 were subsequently visualized by Alexa-568-conjugated anti-mouse and Alexa-647-conjugated anti-rabbit secondary antibodies, respectively. Images were collected with a 63× oil-immersion objective on a Zeiss LSM510 confocal microscope. Fluorescence intensities were quantified using ImageJ software (NIH) for surface and total receptors. Data were expressed as the surface/total AMPAR ratio.

### 4.9. Western blotting

To determine the level of GFP-SFPQ protein expression, we transfected HEK293T cells with pEGFP-SFPQ, either WT, N533H or L534I, for 48 h using the standard calcium phosphate precipitation method. Cells were lysed in 1× SDS sample buffer, resolved on a 10% SDS polyacrylamide gel and analysed by western blotting. Membranes were probed with specific antibodies against GFP (50430-2, Proteintech) and β-actin (clone C4, Santa Cruz Biotechnology) and analysed using the enhanced chemiluminescence method. Images were acquired on the Odyssey Fc imaging system (LI-COR) and band intensities were quantified using Image Studio Lite software (LI-COR).

## Data Availability

Atomic coordinates and structure factors for the reported crystal structures have been deposited with the Protein Data Bank under the accession number 7SP0. The data are provided in electronic supplementary material [65].
